# Probiotics and iron bioavailability: mechanisms, clinical evidence, and translational applications—a comprehensive review of the probiotic strain *Lactiplantibacillus plantarum* 299v (LP299V®)

**DOI:** 10.3389/fnut.2026.1905459

**Published:** 2026-08-07

**Authors:** Brandy Webb, Mary Farrell, Caroline Montelius, Gunilla Önning

**Affiliations:** Probi AB, Lund, Sweden

**Keywords:** Clinical evidence, iron bioavailability, iron deficiency, *Lactiplantibacillus plantarum*, *Lactiplantibacillus plantarum* 299v, microbiota

## Abstract

Iron deficiency remains the most prevalent micronutrient deficiency globally, driven in large part by the limited bioavailability of non-heme iron and compounded by dietary inhibitors, inflammation-mediated hepcidin regulation, and challenges associated with conventional iron supplementation. Health effects of prolonged iron deficiency include heart irregularities, weakened immunity, and cognitive impairment. Growing evidence highlights the gut microbiome as a key modulator of nutrient absorption, with probiotics emerging as a promising adjunctive strategy. Among these, the *Lactiplantibacillus plantarum* strain Lp299v (LP299V®, proprietary strain of Probi®) has demonstrated consistent efficacy in enhancing non-heme iron bioavailability through multiple complementary mechanisms, including luminal acidification via organic acid production, stabilization and chelation of soluble iron complexes, facilitation of ferric-to-ferrous reduction, and potential mitigation of inhibitors such as phytates. Additional effects on host physiology, such as improved intestinal barrier function, increased short-chain fatty acid production, and attenuation of inflammation, further support its role in optimizing iron uptake. The present review gives an overview of how probiotic strains may modulate iron absorption, and more specifically focuses on describing effects obtained with the Lp299v strain. Ten peer-reviewed clinical studies have described the effects of Lp299v for increasing iron absorption. Human isotope studies show that Lp299v increases fractional iron absorption by approximately 20–50% across diverse dietary contexts, while randomized controlled trials and meta-analyses indicate meaningful improvements in iron status, particularly in populations with elevated requirements such as women of reproductive age, pregnant women, and athletes. From the mechanistic studies combined with clinical evidence, results suggest that Lp299v functions as a bioavailability enhancer rather than a substitute for iron intake. Given its versatility in delivery formats, favorable safety profile, and applicability across a range of dietary patterns and socioeconomic settings, Lp299v represents a microbiome-informed approach to addressing the persistent global burden of iron deficiency that has great potential for widespread adoption. This review aims to describe the gut microbiome as an important determinant of micronutrient bioavailability, and, more specifically, to summarize the data supporting Lp299v as a key modulator of iron absorption.

## 1 Introduction

Iron is an essential trace element that plays a central role in numerous physiological processes fundamental to human health. It is a critical component of hemoglobin and myoglobin, enabling oxygen transport and storage, and serves as a cofactor for enzymes involved in DNA synthesis, electron transport, and cellular respiration (1). Given its indispensable biological functions, disruptions in iron homeostasis can have significant clinical consequences. Iron deficiency remains the most prevalent micronutrient deficiency worldwide and is the leading cause of anemia, affecting an estimated 2 billion individuals (2). Populations at greatest risk include women of reproductive age, pregnant women, and young children, largely due to increased iron requirements associated with growth, menstruation, and pregnancy, coupled with inadequate dietary intake or limited access to bioavailable iron sources. Athletes are another at-risk population due to increased iron demand secondary to intense oxidative respiration and ATP needs.

Despite decades of public health efforts, including widespread iron supplementation programs and food fortification strategies, the global burden of iron deficiency has remained persistently high. The limited success of these interventions can be attributed to multiple interacting factors. A key challenge lies in the inherently low bioavailability of non-heme iron, the predominant form of dietary iron consumed globally, especially in plant-based diets. Non-heme iron absorption is highly sensitive to the composition of the diet and is frequently inhibited by compounds such as phytates and polyphenols, which form insoluble complexes with iron in the intestinal lumen (1, 3, 4). In addition to dietary factors, physiological mechanisms also play a critical role. Intestinal hyperpermeability and inflammation reduce intestinal iron absorption and promote sequestration of iron within storage sites, further limiting its availability for erythropoiesis. This is largely thought to be related to increases in hepcidin, a master regulator of iron metabolism and an emerging area of scientific study (5). Together, these barriers significantly constrain the effectiveness of conventional iron interventions.

Oral iron supplementation, while widely used, presents additional limitations that compromise its long-term utility. Gastrointestinal (GI) side effects, including nausea, abdominal discomfort, constipation, and diarrhea, are commonly reported and can substantially reduce adherence, particularly among otherwise healthy individuals receiving preventive supplementation. In fact, up to 60% of people taking oral iron supplements report GI side effects (6). Moreover, unabsorbed iron in the gut lumen may alter the intestinal environment, potentially promoting oxidative stress and unfavorable shifts in microbial composition (7). These factors highlight the need for complementary approaches that improve iron bioavailability while minimizing adverse effects.

In this context, advances in microbiome research have provided new insights into the role of the gut microbial ecosystem in regulating nutrient metabolism and absorption. The intestinal microbiota contributes to host physiology through a range of mechanisms, including modulation of luminal pH, production of metabolites such as short-chain fatty acids, and interaction with epithelial transport systems (8–10). Probiotics, defined as live microorganisms that confer health benefits when administered in adequate amounts (11), are supported by decades of use as modulators of intestinal function. *In vitro* and clinical studies demonstrate that certain probiotic strains can enhance mineral solubility, improve epithelial barrier integrity, and influence host regulatory pathways, thereby potentially increasing the bioavailability of micronutrients such as iron (12–14).

Several probiotics have been clinically studied for their ability to improve iron absorption and iron status, including *Lacticaseibacillus casei* CRL431, heat-killed *Lactococcus lactis* subsp. *cremoris* H61, *Limosilactobacillus reuteri* DSM 17938, and *Lactiplantibacillus plantarum* 299v (Table 1). One clinical trial has investigated *L. casei* CRL431 and found that intake of this strain had no effect on iron status vs. control in healthy children (15). Two clinical trials have been published on heat-killed *L. lactis* subsp. *cremoris* H61 describing beneficial effects on serum iron, transferrin saturation, and ferritin vs. control in healthy women, and serum iron and hemoglobin vs. control in healthy male athletes (16, 17). Three clinical trials have investigated the effects of *L. reuteri* DSM 17938 on iron absorption and iron status in various populations: healthy children, children with iron deficiency, and healthy infants. The study involving healthy children found that intake of this strain had no effect on iron status vs. control (15), whereas in children with iron deficiency, *L. reuteri* DSM 17938 increased reticulocyte hemoglobin equivalent better than the control (18). The third clinical trial investigated effects of *L. reuteri* DSM 17938 when co-administered with human-identical milk oligosaccharides (HiMOs) in healthy infants. In this case, researchers determined that the synbiotic did not affect iron absorption vs. control (19).

**Table 1:** Summary of probiotic strains with human data on iron effects.

Strain	Iron-related clinical trials	Populations studied	Formats studied	Doses studied	Iron-related findings
*L. casei* CRL431 (live)	(15)	Healthy children (1–6 y)	Fortified milk	5 × 10^8^ CFU/d	No change to iron status vs. control in children
*L. lactis* subsp. *cremoris* H61 (heat-killed)	(16, 17)	Healthy women, healthy male athletes	Tablet	30 mg/d; 1.6 × 10^8^ cells/d	↑ SI, TSAT, and SF vs. control in healthy women; ↑ SI and Hb and no change in TIBC vs. control in healthy male athletes
Lp299v (live)	(46–52, 54, 56, 57)	Healthy women, healthy pregnant women, female athletes with low iron stores, adults with IDA, children with ID (5–18 y)	Fermented oat gruel, fermented fruit beverage, freeze-dried powder capsules	1 × 10^9^–2 × 10^10^ CFU/d	↓ IRT-associated GI intolerance and premature IRT discontinuation vs. control in IDA adults; ↑ SI, SF, Hb and ↓ TSAT and TIBC vs. control in IDA adults; attenuated decrease in SF and Hb vs. control in healthy pregnant women; ↓ ID and IDA vs. control in healthy pregnant women; ↑ vigor and VO2max vs. control in healthy female athletes; ↑ FIA vs. control in healthy women; no change to iron status vs. control in ID children
*L. reuteri* DSM 17938 (live)	(15, 18, 19)	Healthy infants (8–14 m), healthy children (1–6 y), children with IDA (5–12 y)	Infant formula, fortified milk, tablet	1 × 10^8^–5 × 10^8^ CFU/d	No change to FIA vs. control in healthy infants; no change to iron status vs. control in healthy children; ↑ Ret-He vs. control in IDA children

ID, Iron deficiency; IDA, Iron deficiency anemia; SF, Serum ferritin; TSAT, Serum transferrin saturation; IRT, Iron replacement therapy; Hb, Serum hemoglobin; SI, Serum iron; TIBC, Total iron-binding capacity, FIA, Fractional iron absorption; Ret-He, Reticulocyte hemoglobin equivalent.

Among the probiotic candidates investigated to date, *Lactiplantibacillus plantarum* 299v (*L. plantarum* 299v, Lp299v, LP299V®) is the most well-studied, with a total of 10 clinical trials. Human studies have shown consistent effects in enhancing non-heme iron absorption. Moreover, several studies have investigated the proposed underlying mechanisms for a probiotic strain to enhance iron absorption. For the Lp299v strain the described mechanisms include the production of organic acids that lower intestinal pH, facilitation of ferric-to-ferrous iron reduction, and mitigation of mucosal inflammation to support more efficient uptake (20). These findings suggest that targeted probiotic interventions may represent a viable strategy to complement existing nutritional approaches in a strain-dependent way. This review summarizes the evidence supporting Lp299v as a modulator of iron absorption and explore its translational potential in addressing the persistent global challenge of iron deficiency.

## 2 Iron absorption and regulation

### 2.1 Iron forms and absorption pathways

Dietary iron exists as two primary forms: heme iron, derived from hemoglobin and myoglobin in animal-based foods, and non-heme iron, which is found predominantly in plant-based sources as well as in fortified foods. Although heme iron is absorbed with relatively high efficiency (approximately 25%) and is less affected by dietary composition, non-heme iron accounts for the majority of global iron intake, particularly in low- and middle-income settings where plant-based diets predominate and may be accompanied by a lack of dietary diversity (21). Despite its abundance, non-heme iron exhibits considerably lower bioavailability (less than 15%) due to its susceptibility to luminal inhibitors and its dependence on specific physicochemical conditions, such as oxidation state, for absorption.

**Figure 1:**
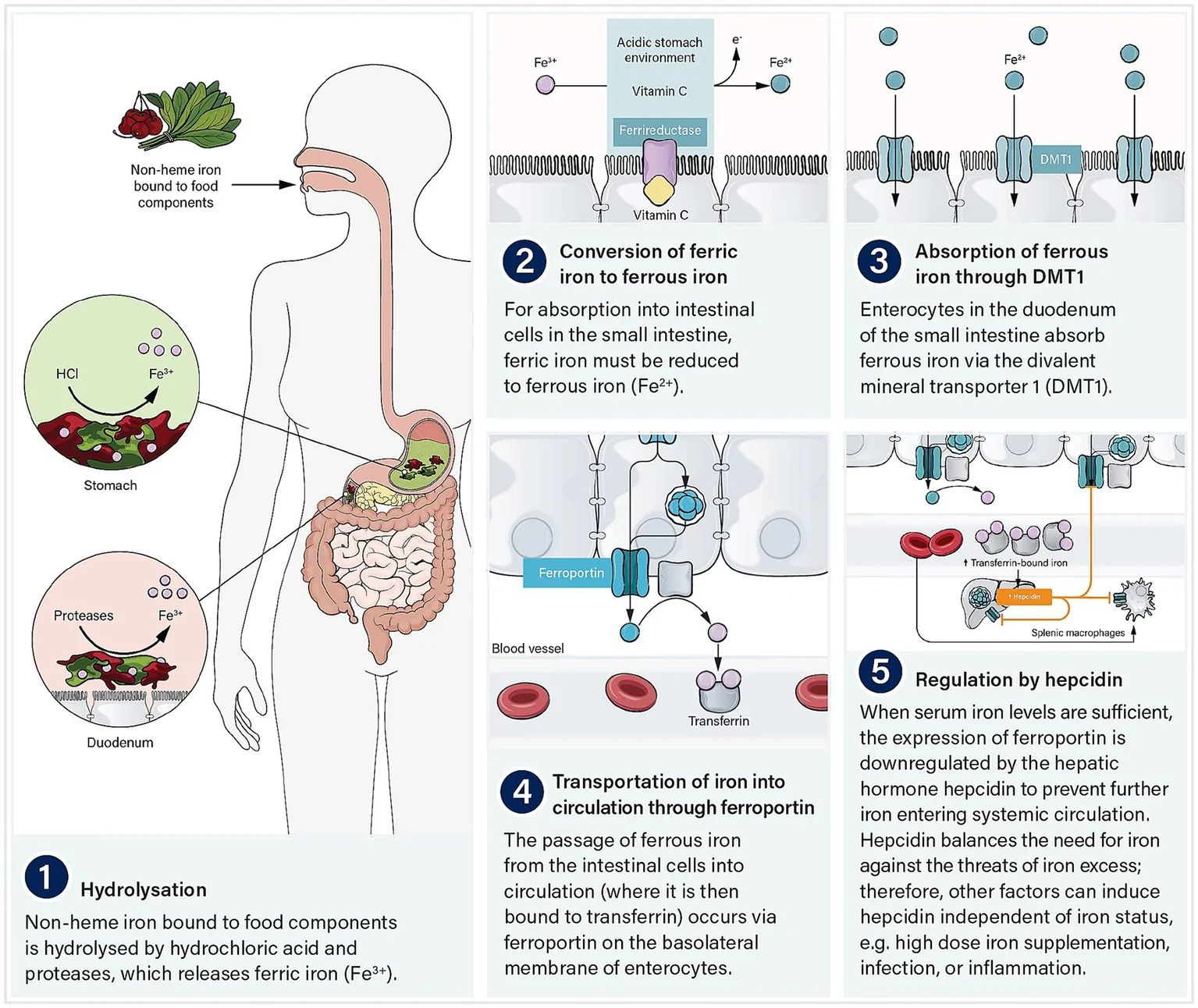
An overview of non-heme iron absorption. Illustration by Melissa Lee, Education Content Specialist, Biome Australia Limited. With permission from ©Biome Australia Limited.

Absorption of non-heme iron occurs primarily in the duodenum and proximal jejunum, where a tightly regulated sequence of biochemical transformations facilitates its uptake (Figure 1). In the intestinal lumen, non-heme iron is typically present in the ferric (Fe^3+^) state, which has poor solubility at physiological pH (22). Prior to uptake, ferric iron must be reduced to the ferrous (Fe^2+^) form by brush border ferrireductases, such as duodenal cytochrome B (DCYTB). This reduction is a critical, rate-limiting step and is strongly influenced by luminal factors including pH and the presence of dietary reducing agents such as ascorbic acid (23, 24).

Once reduced, ferrous iron is transported across the apical membrane of enterocytes via divalent metal transporter 1 (DMT1), a proton-coupled transporter that facilitates the uptake of divalent metal ions. Within the enterocyte, iron enters a metabolically active intracellular pool where it may follow one of two principal fates. It can be sequestered and stored in ferritin, serving as a temporary reserve that is ultimately lost upon sloughing of intestinal epithelial cells, or it can be exported across the basolateral membrane into systemic circulation (25).

Iron export from enterocytes is mediated by ferroportin, the only known cellular iron exporter. Following export, ferrous iron is oxidized back to the ferric state by hephaestin or ceruloplasmin, enabling its binding to transferrin for transport in the bloodstream. This process ensures the safe and efficient delivery of iron to peripheral tissues while minimizing the potential for oxidative damage (26).

Systemic iron homeostasis is tightly regulated by hepcidin, a peptide hormone synthesized predominantly in the liver. Hepcidin exerts its regulatory effects by binding to ferroportin on enterocytes and macrophages, promoting its internalization and degradation, thereby reducing iron efflux into circulation (27). Elevated hepcidin levels—typically observed during inflammation, iron sufficiency, or excess iron replacement—reduce intestinal iron absorption and promote iron sequestration. Conversely, low hepcidin levels enhance ferroportin activity and facilitate increased dietary iron uptake (28); in cases of primary hepcidin deficiency (usually related to autosomal recessive mutations in the hepcidin gene or genes related to hepcidin regulation), potentially life-threatening hemochromatosis, or excess levels of iron in the body, can result (29).

### 2.2 Dietary factors

Iron absorption is strongly influenced by numerous dietary components that act as either enhancers or inhibitors within the gastrointestinal tract. Ascorbic acid is the most potent enhancer, forming soluble iron–ascorbate complexes and reducing ferric iron, thereby increasing absorption up to 2–3 fold in mixed meals (1, 30, 31). In contrast, phytates (found mainly in whole grains and legumes), polyphenols (in tea, coffee, and a variety of fruits, vegetables and berries), and calcium (in dairy, whole grains, fruits and vegetables, and fortified foods) inhibit absorption by forming insoluble complexes or competing for transport pathways (32, 33). Diets relying heavily on these foods without adequate enhancers are therefore associated with significantly higher prevalence of iron deficiency, particularly in low-income populations (34).

Additionally, at the systemic level, dietary iron intake interacts with the hepcidin–ferroportin axis. Diet-induced changes in circulating iron and inflammation modulate hepcidin expression; for instance, iron-rich meals acutely increase hepcidin levels within hours, transiently reducing subsequent absorption. Conversely, iron deficiency and hypoxia suppress hepcidin, enhancing iron uptake (29).

Epidemiologically, diet quality strongly correlates with iron status outcomes. Populations consuming predominantly plant-based diets with low bioavailability exhibit iron deficiency prevalence rates exceeding 30–40%, compared with <10% in populations with higher intake of heme iron from meat sources (35, 36). Additionally, specific physiological states (e.g., menstruation, pregnancy, and intensive exercise) increase iron needs and thus susceptibility to deficiency, particularly when dietary iron density is inadequate. For example, pregnant women require approximately 27 mg/day of iron, yet typical intake in many regions is <15 mg/day (37, 38). These mismatches between intake, bioavailability, and physiological need highlight the importance of both dietary composition and total intake in determining deficiency risk.

Overall, diet influences iron status through a multifactorial network involving chemical speciation, luminal interactions, intestinal transport mechanisms, and systemic regulatory pathways. Strategies to reduce iron deficiency therefore include increasing intake of bioavailable iron (e.g., animal-source foods), enhancing absorption through dietary modifiers (e.g., vitamin C-rich foods), reducing inhibitors (e.g., phytates and polyphenols), and implementing fortification or supplementation programs in high-risk populations. One adjunctive approach gaining recognition is the use of microbiome-targeting therapies such as Lp299v to increase non-heme dietary and supplemental iron bioavailability.

### 2.3 Iron status evaluation and diagnosis

Serum-based biomarkers are commonly used in combination to characterize iron status. Serum ferritin is the primary indicator of iron stores, with low concentrations reflecting depleted body iron, although it must be interpreted wholistically as it can rise during times of inflammation or infection and potentially mask a deficiency (39). Soluble transferrin receptor (sTfR) reflects cellular iron demand and erythropoietic activity, increasing in states of iron-deficient erythropoiesis and remaining relatively unaffected by inflammation, which enhances its utility in distinguishing iron deficiency from anemia of inflammation (39). Total body iron, a calculated value based on the ratio of sTfR to ferritin, integrates information on iron stores and tissue iron demand, providing a continuous measure across the full iron status spectrum and improving diagnostic accuracy (40). Hemoglobin (Hb) is the most widely used clinical indicator of iron deficiency anemia, although it reflects a late and nonspecific stage of deficiency; reticulocyte hemoglobin content offers a more sensitive measure of recent iron availability for erythropoiesis (41, 42). Transferrin saturation (TSAT), calculated as the ratio of serum iron to total iron-binding capacity, reflects circulating iron available for erythropoiesis and is typically reduced in iron deficiency (43). Serum iron represents circulating transferrin-bound iron; however, it is highly variable due to diurnal rhythms, dietary intake, and inflammation, limiting its standalone diagnostic value. Thus, serum iron is best interpreted alongside other iron status markers such as ferritin and TSAT (39).

Diagnostic criteria for iron deficiency and iron deficiency anemia (IDA) are typically defined using combinations of these biomarkers. Iron deficiency is generally indicated by depleted iron stores, most commonly assessed as low serum ferritin (e.g., <15–30 μg/L, depending on population), often accompanied by reduced TSAT and elevated sTfR (39, 43). IDA represents a more advanced stage characterized by impaired erythropoiesis, defined by low Hb concentrations (e.g., <13 g/dL, depending on population), alongside low serum ferritin, with additional features such as low TSAT or elevated sTfR (44). Because individual biomarkers have inherent limitations, current guidelines emphasize the use of multiple indicators and context-specific thresholds to accurately diagnose iron deficiency and IDA across clinical and population settings (39).

## 3 Lp299v-driven Iron absorption

Given the complexities of iron homeostasis in humans and the need for iron deficiency therapies, comprehensive research efforts have been undertaken to investigate Lp299v’s potential as an iron support adjunctive therapy, spanning *in vitro* experiments, absorption studies, and long-term intervention trials (Table 2).

**Table 2:** Summary of clinical trials involving Lp299v and iron absorption or iron status.

Author, year	Country	Design	Subjects	Intervention	Key outcomes
Axling et al. (54)	Sweden	Double-blind, randomized, controlled	53 healthy, non-anemic female athletes with low iron stores	10B CFU Lp299v + 20 mg iron capsule daily; 12 weeks	SF + 13.6 μg/L (+70%) vs. + 8.2 μg/L (+42%) at week 4 (*p* = 0.056); Ret-Hb + 1.5 vs. + 0.82 pg. (*p* = 0.083); Vigor +3.5 vs. + 0.1 (*p* = 0.015); VO2max ↑ (*p* < 0.05 for all timepoints)
Axling et al. (46)	Sweden	Double-blind, randomized, placebo-controlled	326 pregnant women	20B CFU Lp299v + 4.2 mg iron, 30 µg folic acid, and 12 mg ascorbic acid capsule daily; 10–12 GW to delivery	SF decline attenuated at week 28(*p* = 0.003), and week 35 (*p* < 0.001); SF week 35: 16.0 vs. 13.8 μg/L (*p* < 0.001); ID 59% vs. 78% (*p* = 0.017); IDA 7.4% vs. 21% (*p* = 0.023); Hb week 35: 122.5 vs. 117.4 g/L (*p* = 0.002)
Bering et al. (48)	Denmark	Double-blind, cross-over	24 healthy, non-anemic women	100B CFU Lp299v in fermented oat gruel daily; 4 days	Iron absorption 1.1% vs. 0.5–0.6% (*p* < 0.0001); + ~ 80% vs. control
Bering et al. (50)	Denmark	Double-blind cross-over	18 healthy, non-anemic women	100B CFU Lp299v lyophilized powder; 4 days	Iron absorption 1.4% vs. 1.3% (ns); Distal intestine absorption <0.1%
Hemphill et al. (52)	United States	Double-blind, randomized, placebo-controlled	20 (13 completed) pregnant women	10B CFU Lp299v + 27 mg iron capsule daily; 15–20 GW to delivery	Maternal hematologic and iron parameter decline attenuated vs. placebo; IDA at delivery: 1/5 vs. 2/4 (per-protocol); Neonatal SI 136.2 vs. 108.0 μg/dL Neonatal TSAT 63.8% vs. 43.3% (ns)
Hoppe et al. (49)	Sweden	Single-blind, cross-over	(1) 10 healthy women (2) 11 healthy women	(1) 10B CFU Lp299v in fruit drink daily; 4 days (2) 1B CFU Lp299v in fruit drink daily; 4 days	Trial 1: 28.6% vs. 18.5% Iron absorption (+54.6%), (*p* = 0.028); Trial 2: 29.1% vs. 20.1% (+44.8%), (*p* = 0.080); Pooled data: 28.8% vs. 19.3% (*p* = 0.004); + ~ 50% vs. control
Hoppe et al. (51)	Sweden	Single-blind, cross-over	(1) 14 healthy women (2) 28 healthy women	10B CFU Lp299v + 4.2 mg iron, 30 μl folic acid, and 12 mg ascorbic acid daily; 4 days	Trial 1: 22.4% vs. 17.4% Iron absorption (+28.7%), (*p* = 0.040); Trial 2: 24.5% vs. 20.9% (+17.2%), (*p* = 0.003); Pooled data: 23.8% vs. 19.7% (p < 0.05); + ~ 20% higher vs. control
Koker et al. (47)	Turkey	Randomized, controlled	295 newly diagnosed IDA subjects starting high-dose IRT	10B CFU Lp299v + 100 mg iron capsule daily; 12 weeks	GI intolerance 13.0% vs. 46.5% (*p* < 0.001); Treatment discontinuation within 30 days 3.6% vs. 15.9% (*p* < 0.001); SI 76 vs. 60 μg/dL (*p* < 0.001); TSAT 20.1% vs. 14.5% (*p* < 0.001), Hb + 0.9 vs. + 0.4 g/dL (*p* < 0.001)
Korčok et al. (56)	Serbia	Double-blind, randomized, controlled	20 healthy women	10B CFU Lp299v + 10 mg sucrosomial iron + 15 mg vitamin C capsule daily; 7 days	SI 19.4 vs. 17.5 μmol/L (+ ~ 11%)
Rosen et al. (57)	United States	Double-blind, randomized, controlled	52 children with ID	10B CFU Lp299v + 1–3 mg/kg/day iron	SF: 23.2 vs. 20.0 ng/mL control (ns)

ID, Iron deficiency; IDA, Iron deficiency anemia; SF, Serum ferritin; SI, Serum iron; TSAT, Serum transferrin saturation; Hb, Serum hemoglobin; Ret-He, Reticulocyte hemoglobin equivalent, GW, Gestational weeks, IRT, Iron replacement therapy.

### 3.1 *In vitro* data

The influence of Lp299v on iron bioavailability was studied using simulated gastrointestinal digestion combined with a human intestinal co-culture model (Caco-2/HT29-MTX), where it was shown that inclusion of Lp299v in iron-containing foods alters iron chemistry during digestion, specifically increasing the level of ferric iron by between 16 ± 1.49% and 39 ± 1.0%, depending on the delivery format (capsule vs. functional drink; *p* < 0.05 vs. control for all formats) (45).

When these Lp299v-containing digests were applied to intestinal epithelial cells, this resulted in a targeted cellular response: expression was increased of DCYTB (*p* < 0.05), the enzyme critical for converting ferric iron to ferrous iron, the form that can be transported into enterocytes. However, the membrane transporter DMT1 was not clearly upregulated, which may indicate that the primary effect is on the initial reduction step rather than the downstream transport step (45). While definitive DMT1 data is lacking, clinical trials suggest that the increased ferrous iron pool moves from the intestinal lumen into the enterocytes and ultimately into the bloodstream, as evidenced by increased iron status markers such as serum ferritin and serum hemoglobin (46, 47). Moreover, the meal-absorption studies by Bering (48) and Hoppe et al. (49) further show increased retention of the isotope-labeled iron in blood samples, further demonstrating that the absorbed iron moves from the enterocytes into the blood stream. It can be speculated, then, that either (1) ferrous iron relies on homeostatic DMT1 transporter dynamics to enter the intestinal cells in the absence of Lp299v up regulatory mechanisms, or (2) upregulation of DMT1 occurs, but this effect has not been properly characterized.

### 3.2 Human Iron absorption meal studies

Early controlled, crossover studies provide the clearest demonstration that Lp299v can enhance iron uptake from meals. These controlled human isotope studies demonstrated that Lp299v enhances non-heme iron absorption under defined dietary conditions. They used validated double-isotope methodologies (^55^Fe/^59^Fe) with normalization to a 40% reference dose to account for inter-individual variation in iron status. The first study investigated an oat gruel fermented with live Lp299v and showed significantly increased non-heme iron absorption from a phytate-rich meal in women with low iron stores, approximately doubling absorption compared with pasteurized or non-fermented controls. The effect was specific to live bacteria and could not be explained solely by organic acids or pH, suggesting a biological role of Lp299v itself in facilitating iron uptake (48). A subsequent study by the same researchers investigating lyophilized Lp299v did not show similar results (50). In this study lyophilized Lp299v was added directly to a heat-treated, Lp299v-fermented oat gruel with a low pH. To explain the absence of an effect on iron absorption, the activity of the lyophilized Lp299v was investigated (50). The results indicated that, when added in this manner, Lp299v required a longer time to become metabolically active, which may have reduced its potential to influence iron absorption. Additionally, they were particularly interested in investigating whether the large intestine could be a site of iron absorption, but the data did not support this hypothesis.

The study by (49), included women of reproductive age who consumed an iron-fortified fruit drink with relatively high baseline bioavailability. This study showed that the mean fractional iron absorption increased from 19.3 ± 6.0% to 28.8 ± 14.7% with the addition of Lp299v (~50% relative increase; *p* < 0.004). This effect was observed at both 10^9^ and 10^10^ CFU of Lp299v without a clear dose–response (49). Complementary findings were reported by the same researchers in two sequential single-blind studies in women of reproductive age, in which co-administration of 10^10^ CFU lyophilized Lp299v included in a capsule with a standardized wheat-based meal increased iron absorption from 17.4 ± 13.4% to 22.4 ± 17.3% (*p* = 0.04) and from 20.9 ± 13.1% to 24.5 ± 12.0% (*p* = 0.003), corresponding to a ~ 17–29% relative increase (51). Collectively, these data indicate a reproducible enhancement of fractional non-heme iron absorption by Lp299v across different delivery formats (food matrix and capsule) and dietary contexts (varying levels of dietary enhancers and inhibitors).

### 3.3 Long-term Iron status trials

More recent randomized, controlled trials suggest that the above described absorption effects translate into clinical improvement of iron status. In a randomized, double-blind, placebo-controlled trial involving 326 healthy pregnant women beginning at gestational weeks 10–12, Lp299v (10^10^ CFU/day), combined with 4.2 mg iron, 12 mg ascorbic acid, and 30 μg folic acid was dosed twice daily. Results showed significant improvement in multiple indices of iron status when compared to placebo, Specifically, Lp299v attenuated the physiological decline in serum ferritin during pregnancy compared with placebo (p = 0.003 at gestational week 28; *p* < 0.001 at gestational week 35), reflecting improved maintenance of iron stores. This effect extended to functional outcomes, including a significantly lower prevalence of iron deficiency (59% vs. 78%, *p* = 0.017) and iron deficiency anemia (7.4% vs. 21%, *p* = 0.023) at 35 weeks’ gestation compared to the placebo group. Additional mechanistic biomarkers supported enhanced iron utilization, as evidenced by increased soluble transferrin receptor (*p* = 0.011) and a reduced decline in total body iron (p < 0.001). These findings suggest that Lp299v enhances iron bioavailability sufficiently to slow depletion of maternal iron stores, likely through improved intestinal uptake and systemic distribution of dietary iron (46).

Further evidence of prenatal benefit comes from a double-blind, controlled trial involving 13 pregnant individuals of various socio-economic settings at risk of iron deficiency anemia (defined as Hb 10–12 g/dL), which compared supplementation with Lp299v (10^10^ CFU/day alongside an oral prenatal vitamin containing 27 mg iron) to placebo alongside the oral prenatal vitamin intake, from early gestation (15–20 weeks) through delivery. Although not powered for statistical significance, a clear limitation of the study, this pilot trial provided some indicators of attenuation in the decline of hematological and iron status parameters across individuals in the Lp299v group compared with placebo (52). Specifically, trajectories of hemoglobin, ferritin, and transferrin saturation indicated improved maintenance of iron status, with a favorable pattern of increased or stabilized hemoglobin in the Lp299v group versus declining levels in the placebo. Additionally, neonatal outcomes suggested improved iron transfer, as infants in the Lp299v group exhibited higher serum iron (mean 136.2 μg/dL vs. 108.0 μg/dL) and transferrin saturation levels (mean 63.8% vs. 43.3%) at birth, despite mothers starting from a relatively worse baseline iron status (52). Although from a limited pilot study, these findings suggest that Lp299v may enhance not only maternal absorption but also maternal–fetal iron trafficking, likely via improved systemic iron availability. Early iron deficits in neonates are associated with increased risk of cognitive delays and immune dysfunction, underscoring the importance of supporting iron status in expectant mothers (53).

Additional iron status evidence is provided by a randomized, controlled trial involving 39 female athletes with low iron stores, where Lp299v (10^10^ CFU/day) co-administered with oral iron (20 mg/day) resulted in numerically greater improvements in iron biomarkers. Ferritin increased more rapidly with Lp299v than with iron alone (13.6 vs. 8.2 μg/L, *p* = 0.056) after 4 weeks, and reticulocyte hemoglobin content (a sensitive marker of early erythropoietic iron incorporation) showed a greater increase (1.5 vs. 0.82 pg., *p* = 0.083) after 12 weeks (54). Subgroup analysis further demonstrated a significantly greater ferritin increase in participants with higher baseline iron stores (*p* = 0.0361) (54), which is consistent with findings that in individuals with greater iron depletion, erythropoiesis is prioritized over iron storage to meet immediate iron needs, thus ferritin levels are slower to increase (55). These results did not reach conventional significance thresholds for primary endpoints, although they show consistent directional improvements across multiple markers and may suggest a biologically meaningful effect on iron absorption and utilization. Indeed, participants in the Lp299v group reported significantly increased vigor compared to the placebo group after 12 weeks (*p* = 0.015) (54).

Further evidence comes from a randomized trial involving 295 patients with newly diagnosed IDA, in which adjunctive Lp299v supplementation (10^10^ CFU/day) significantly improved hematologic and biochemical iron parameters during oral iron therapy (100 mg/day). Compared with iron supplementation alone, the Lp299v group exhibited greater increases in serum iron (23.5 vs. 8.0 μg/dL, *p* < 0.001), transferrin saturation (8.2% vs. 2.1%, *p* < 0.001), ferritin (13.0 vs. 5.0 ng/mL, *p* < 0.001), and hemoglobin (0.9 vs. 0.4 g/dL, *p* < 0.001) after 3 months intervention (47). These improvements occurred alongside enhanced treatment adherence driven by reduced gastrointestinal intolerance, suggesting a dual mechanism whereby Lp299v not only increases iron absorption at the intestinal level but also improves effective iron repletion by enabling sustained therapy.

The clinical trials described thus far involve supplemental ferrous fumarate, one of the most prescribed iron salts. Today, supplemental iron is available in a variety of forms, including liposomal and sucrosomal forms, which are characterized by better bioavailability and fewer gastrointestinal side effects. In a controlled clinical intervention trial, co-administration of Lp299v with sucrosomal iron and vitamin C resulted in measurable improvements in circulating iron markers relative to iron supplementation alone. Following a 7-day supplementation period in 20 healthy women of reproductive age, serum iron concentrations were higher in the Lp299v group compared with control (19.4 vs. 17.5 μmol/L), corresponding to an approximate 11% higher level, attributed to enhanced intestinal absorption (56). One limitation of this publication is the lack of a formal statistical analysis. However, the consistent directional improvements across multiple biomarkers (including ferritin and hemoglobin) support a mechanistic effect of Lp299v on iron uptake and expand the pool of evidence to include alternate forms of iron with characteristic bioavailability and GI tolerance profiles.

While the majority of longitudinal human trials demonstrate iron absorption effects with Lp299v, one study from 2019 failed to show significant results. In a study involving 43 iron-deficient children with restless sleep, aged 5–18 years, researchers investigated the effect on iron status of consuming iron (3 mg/kg/day) and vitamin C (125 mg/day or 250 mg/day, depending on age) plus either Lp299v (10^10^ CFU/day) or placebo. After 6–8 weeks of treatment, the Lp299v group tended toward higher serum ferritin levels compared with placebo (23.2 ng/mL vs. 20.0 ng/mL); however, these results did not reach statistical significance. The authors identify several limitations of the study that could potentially explain the results, particularly around heterogeneity of iron dose and duration, and recommend further investigation (57).

On the whole, the clinical trials described herein reinforce the broader evidence base demonstrating that defined probiotic strains, such as the *L. plantarum* strain Lp299v, can improve iron status through multifactorial mechanisms centered on enhanced intestinal absorption and improved systemic handling of iron. Lp299v findings were validated in a 2025 systemic review and meta-analysis, which concluded that Lp299v demonstrated a consistent and clinically meaningful role in enhancing iron absorption and improving iron stores (58). Specifically, the authors concluded that evidence for Lp299v’s role in iron nutrition was strongest for improving bioavailability and absorption, moderate for increasing iron stores, and limited for impacting hemoglobin. This reaffirms Lp299v primarily as a bioavailability enhancer, improving the efficiency of iron utilization and contributing to better iron status over time, particularly when paired with dietary or supplemental iron.

## 4 Lp299v mechanisms of action

**Figure 2:**
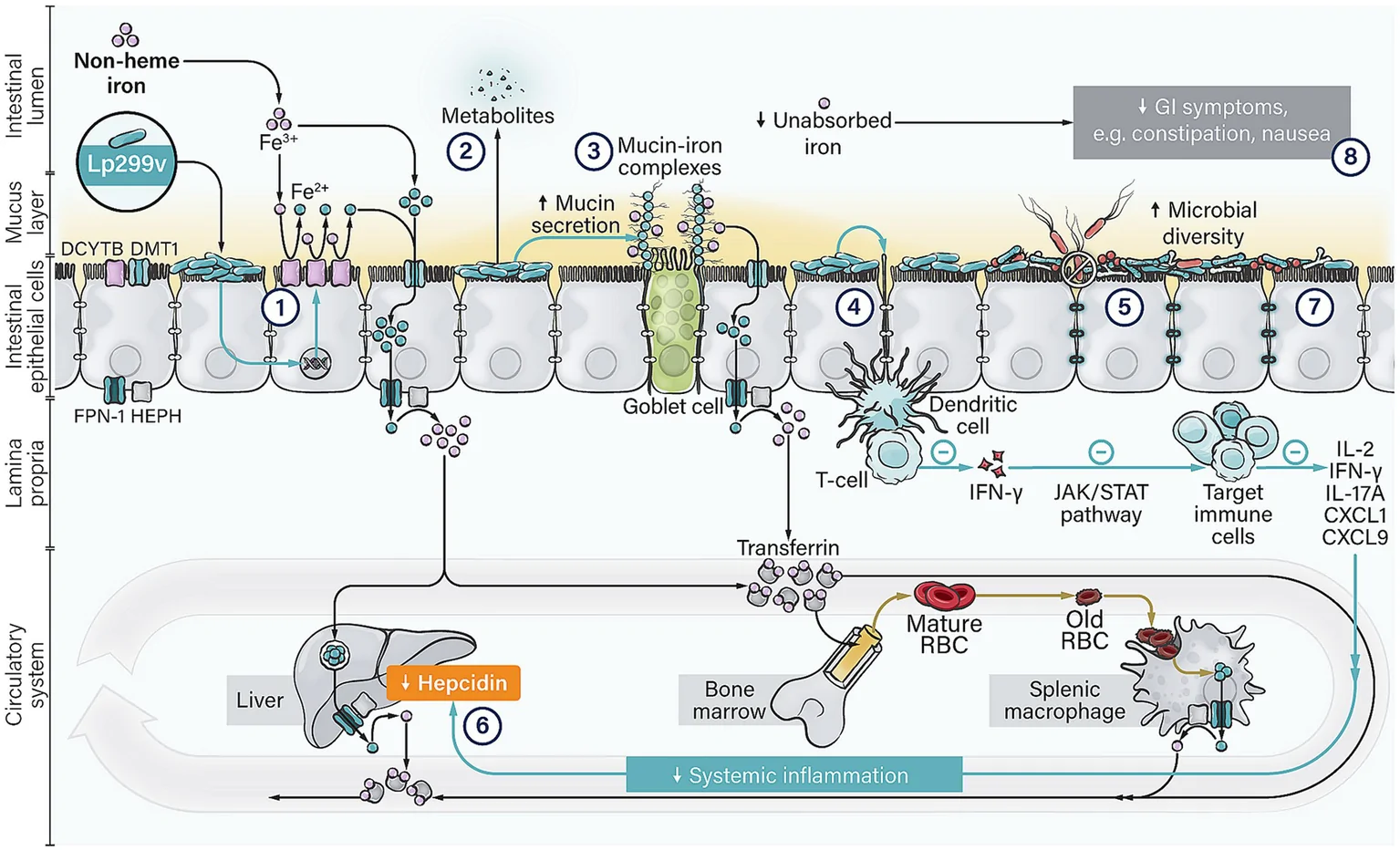
Mechanistic overview of Lp299v effects on iron absorption. (1) Lp299v upregulates the expression of the ferrireductase enzyme DCYTB, thereby increasing the conversion of ferric iron to ferrous iron for transportation into intestinal epithelial cells via DMT1. (2) Lp299v produces metabolites including SCFAs that affect lumen pH, barrier integrity, and inflammatory pathways, impacting iron absorption. (3) Enhanced mucin production by Lp299v may lead to the formation of mucin-iron complexes that support barrier integrity and iron absorption. (4) Lp299v downregulates IFN-*γ*-driven JAK/STAT pathways in target immune cells, reducing pro-inflammatory compounds like IL-2, IFN-γ, IL-17A, CXCL9, and CXCL1, thus reducing systemic inflammation. (5) Lp299v promotes intestinal mucosal health by increasing microbial diversity, enhancing tight junction integrity, and helping to reduce inflammation caused by bacterial translocation and immune activation. (6) By reducing inflammation, Lp299v likely helps reduce hepcidin production, allowing more iron to pass through ferroportin on enterocytes, hepatocytes, and splenic macrophages, and enter systemic circulation. (7) Supporting tight junction integrity and reducing inflammation helps promote healthy intestinal villi and brush border membrane function. (8) By enhancing iron uptake from the intestinal lumen, Lp299v reduces common GI side effects associated with iron supplementation. Illustration by Melissa Lee, Education Content Specialist, Biome Australia Limited. With permission from ©Biome Australia Limited.

There are several proposed mechanisms underlying Lp299v’s iron enhancing properties (Figure 2). These span metabolite-driven pH lowering characteristics, ferrireductase upregulation, inflammation modulation, and intestinal barrier strengthening. These are distinct yet interconnected pathways that collectively contribute to the observed clinical benefits.

### 4.1 Luminal acidification and Iron chelation

Lp299v belongs to the group of lactic acid-producing bacteria that ferment carbohydrates into lactic acid and other organic acids including short-chain fatty acids (SCFAs), affecting levels both directly and indirectly via shifts in the overall microbiota milieu. Production of these metabolites is intrinsically associated with a reduction in environmental pH, thereby creating conditions favorable for commensal microbiota while inhibiting pathogens (59). In fermentation models using Lp299v, progressive lactic acid formation is accompanied by measurable decreases in pH (e.g., reductions below pH ~ 4.6 during substrate fermentation), directly demonstrating the strain’s ability to acidify its environment (60). This type of *in vitro* data provides mechanistic validation that the metabolic outputs of Lp299v are sufficient to drive substantial pH reduction under physiologically relevant conditions.

This effect on organic acid level has profound implications for mineral bioavailability, particularly for non-heme iron, which is highly sensitive to changes in chemical speciation within the intestinal environment. Under neutral or slightly alkaline conditions, non-heme iron tends to undergo hydrolysis and precipitate as insoluble iron hydroxides and iron-phytate salts, which are poorly absorbed by enterocytes. Conversely, the decrease in luminal pH induced by Lp299v-derived organic acids shifts the equilibrium toward more soluble ionic forms of iron by forming soluble iron-organic acid complexes, enhancing iron’s chemical stability and availability for uptake (32, 33). This is particularly important in the upper small intestine, where iron absorption primarily occurs and where subtle variations in solubility can significantly influence uptake efficiency. By stabilizing iron in solution, organic acids effectively increase the pool of luminal iron available for absorption via enterocyte iron transport proteins.

### 4.2 Dietary phytase enhancement

Phytate (myo-inositol hexaphosphate), a principal storage form of phosphorus in plant seeds, grains, and legumes, is well recognized as a potent inhibitor of iron absorption. It exerts its inhibitory effect through strong chelation of non-heme iron, forming insoluble iron–phytate complexes that are not readily absorbed in the small intestine. This interaction occurs even at relatively low phytate concentrations and is particularly problematic in diets where phytate-rich staple foods constitute a large proportion of total caloric intake (44, 61–63).

A more acidic environment, aided by lactic acid-producing bacteria such as Lp299v, has been reported to promote phytase activity in foods (64). In an *in-vitro* study investigating the effects of Lp299v fermentation of phytate-rich foods, Castro Alba et al., suggested that Lp299v may indirectly enhance phytate degradation by lowering pH and creating conditions favorable for phytate degradation, thereby resulting in lower iron-chelating capacity and increased iron bio-accessibility. While the authors stated that phytate degradation was most likely driven by endogenous plant phytases, fermentation of pseudocereal flours with Lp299v did significantly improve iron bio-accessibility in an *in-vitro* digestion model (65). These findings suggest that Lp299v can improve iron bio-accessibility in phytate rich foods and may indirectly enhance endogenous phytase activity via acidification.

### 4.3 Membrane protein modulation

Available evidence suggests that Lp299v may modulate host iron absorption not only through acidification, chelation, and phytase effects, but also via direct influences on epithelial proteins. One proposed pathway involves converting ferric iron into a ferrous form that is amenable to transport at the brush border. Experimental studies indicate that Lp299v can alter the luminal iron pool, including increasing the proportion of absorbable iron species, thereby enhancing substrate availability for epithelial uptake pathways.

To understand how Lp299v achieves this increase in iron reductive capacity, it’s helpful to note that as a mucosa-adherent strain, Lp299v is capable of colonizing and persisting on intestinal epithelial surfaces—including of the small intestine—in humans, as shown *in vitro* and by biopsy-based detection after oral administration (66–69). This adherence within the small intestine is mechanistically important, as close contact with the epithelium enables direct modulation of mucosal membrane proteins in the precise location where iron absorption occurs (70).

A key step in non-heme iron absorption is the reduction of ferric iron to ferrous iron by DCYTB, the key ferrireductase localized on the apical membrane of enterocytes found at the brush border. This reduction is essential for subsequent transport via DMT1, which preferentially imports ferrous iron into the cell (71, 72). Notably, mechanistic studies using Caco-2/HT29 intestinal co-cultures have demonstrated that exposure to Lp299v upregulates the expression of DCYTB, suggesting it enhances the reductive capacity of the intestinal epithelium (45, 73). Given that DCYTB activity is a critical determinant of iron uptake efficiency, this upregulation provides an important mechanistic link between Lp299v and improved iron absorption.

### 4.4 SCFA production and barrier function

Lp299v contributes to improved iron absorption through mechanisms that include enhancement of SCFA production and strengthening of intestinal barrier function. These processes are closely related and increasingly recognized as key determinants of iron bioavailability.

Evidence from both animal and human studies indicates that Lp299v supplementation can increase circulating or luminal SCFAs, including acetate, propionate, and butyrate. These metabolites function as central regulators of intestinal physiology, serving as an energy source for colonocytes and modulating epithelial differentiation, immune signaling, and mucosal homeostasis (74, 75). Of particular interest, butyrate enhances tight junction assembly and epithelial integrity, thereby reinforcing the mucosal barrier and limiting paracellular permeability (9). In a rodent model, dietary supplementation with Lp299v resulted in elevated plasma propionate and butyrate levels, which were associated with enrichment of SCFA-producing taxa such as *Bifidobacteriaceae* and *Clostridiales* (76). These findings were corroborated in a related study showing that Lp299v increases the SCFAs propionate and butyrate and promotes regulatory signaling pathways (77).

A human trial showed that Lp299v consumption for 3 weeks resulted in increased concentration of acetate and propionate (78). In a six-week clinical trial, Lp299v supplementation led to a significant increase in plasma propionate concentrations (79). A separate study involving antibiotic-treated individuals showed that Lp299v helped preserve SCFA levels compared to placebo. Specifically, total SCFAs significantly decreased in the placebo group during metronidazole therapy (77.1 to 45.5 μmol/g; *p* = 0.028), whereas this decline was attenuated in the Lp299v group (79.8 to 60.4 μmol/g), indicating maintenance of colonic fermentation. Notably, butyrate concentrations were significantly better preserved in the Lp299v group compared with placebo during antibiotic exposure (5.6 to 1.2 μmol/g in placebo vs. 7.6 to 5.6 μmol/g in Lp299v; *p* = 0.047), suggesting protection of butyrate-producing microbial activity (80). Collectively, these data suggest that Lp299v may enhance SCFA availability either directly or via microbiome restructuring.

Multiple controlled studies provide direct evidence that Lp299v reduces or prevents increases in intestinal permeability, particularly under conditions of stress or pathogen exposure. In a well-characterized rat model using Ussing chamber methodology, pretreatment with Lp299v abolished the increase in intestinal permeability induced by *Escherichia coli*, as measured by translocation of radiolabeled mannitol across the intestinal epithelium (81). A follow-up study by the same researchers confirmed these results: decreased translocation was observed with Lp299v treatment in a septic rat model (82). Earlier studies were consistent with these findings, including a study in which Lp299v significantly reduced translocation in rats with methotrexate-induced enterocolitis. In this model, the mucosa became inflamed and damaged, and the administration of Lp299v mitigated the mucosal injuries induced by the chemotherapy (83). In a separate study evaluating intestinal mucosa using the content of rRNA and DNA as markers, an improvement in mucosal status was found in rats with acute liver injury that had been pre-treated with Lp299v (84, 85).

Mechanistic studies provide further evidence of Lp299v’s intestinal barrier supporting function, in this case by promoting a robust mucus layer. The intestinal mucus layer, frequently termed the “first line of defense” within the GI tract, is a critical determinant of barrier integrity, forming a biophysical and biochemical interface that segregates luminal pathogens from the epithelium (86). Mucin-2 (MUC2) is a secreted, gel-forming mucin polymer that makes up the majority of the protective mucus layer of the intestinal tract, while mucin-3 (MUC3) is a membrane-bound mucin expressed on enterocyte apical surfaces that acts as a physical shield while influencing intracellular signaling pathways that regulate barrier function (86, 87). MUC2 and MUC3 are both essential for maintaining a healthy gut barrier; indeed, reductions in these compounds lead to mucus layer thinning, which predisposes to hyperpermeability and inflammation (87, 88). Additionally, mucin itself has been shown to play a direct role in facilitating iron absorption through the formation of iron-mucin complexes (89–91). In this context, it is significant that *in vitro* studies involving HT-29 intestinal cells—widely used to investigate gut barrier and mucin behavior—demonstrated that Lp299v rapidly upregulated mRNA expression of the mucin genes *MUC2* and *MUC3*, which was followed by a marked increase in mucin production (92, 93).

### 4.5 Inflammation modulation

Chronic inflammation is well established to impair iron absorption and utilization, largely through cytokine-driven upregulation of hepcidin, the key regulator of systemic iron homeostasis. Pro-inflammatory cytokines—particularly IL-6, but also IL-1β and TNF-*α*—stimulate hepatic hepcidin expression, which in turn reduces intestinal iron absorption and traps iron within macrophages (94–96). Specifically, elevated hepcidin levels promote internalization and degradation of ferroportin, thereby limiting the transfer of iron from intestinal cells into circulation. In inflammatory states, such as infection, chronic disease, or gut dysbiosis, this pathway leads to decreased dietary iron absorption and increased iron sequestration (71). In this context, even modest reductions in inflammatory tone can translate into meaningful improvements in iron bioavailability.

In an *ex vivo* study using human colonic mucosa, Lp299v showed strong anti-inflammatory effects following induced immune activation. Lp299v significantly downregulated key pro-inflammatory cytokines and chemokines, including IL-2, IFN-*γ*, IL-17A, CXCL9, and CXCL11, all of which were highly upregulated during T-cell–mediated inflammation (97). These changes indicate that Lp299v suppresses T-cell activation and proliferation and dampens both Th1 and Th17 immune responses. Lp299v also reduced chemokines involved in immune cell recruitment, suggesting a role in limiting inflammatory cell infiltration into intestinal tissue. Mechanistically, its effects are linked to downregulation of the IFN-γ–driven JAK/STAT signaling pathway, implying upstream modulation of immune signaling networks (97).

Evidence from a human interventional study along with follow-up analyses is consistent with these *ex vivo* data, showing consistent Lp299v-mediated reductions in circulating inflammatory mediators. In men with stable coronary artery disease, 6 weeks of Lp299v supplementation significantly reduced pro-inflammatory biomarkers, including IL-8 (*p* = 0.01), IL-12 (*p* = 0.02), and leptin (*p* = 0.0007). These findings were reinforced by mechanistic analyses showing a marked reduction in a composite inflammatory index (1.01 ± 0.74 vs. 0.22 ± 0.51; *p* < 0.0001) and downregulation of key inflammatory pathways, including IL-1β and interferon signaling (79, 98, 99). Such clinical evidence indicates that Lp299v supplementation attenuates IL-6–related signaling and toll-like receptor activation in humans, pathways that are tightly coupled to hepcidin induction. Additionally, reductions in leptin observed with Lp299v supplementation are relevant to iron metabolism, as leptin itself has been shown to directly upregulate hepcidin, as well as to stimulate inflammatory cytokine production (including of IL-6), thereby potentially further contributing to hepcidin upregulation indirectly (100, 101).

By attenuating cytokine signaling upstream of hepcidin, Lp299v may create a physiological state that permits more efficient dietary iron absorption and systemic utilization, thereby linking its immunomodulatory and nutritional effects.

### 4.6 Gastrointestinal tolerance

The myriad mechanisms by which Lp299v increases iron absorption have a secondary clinical benefit: mitigation of GI intolerance symptoms that frequently accompany iron replacement. Oral iron supplementation is known to leave a proportion of unabsorbed iron in the gut lumen, where it can catalyze the formation of reactive oxygen species, disrupt microbial balance, and induce mucosal inflammation and epithelial injury. Consequently, individuals requiring iron replacement often prematurely terminate their iron supplementation (102). By reducing the amount of iron that remains in the lumen, Lp299v helps mitigate this localized oxidative and inflammatory stress. Gut barrier strengthening, SCFA production, and inflammation modulation by Lp299v further aid in limiting mucosal damage and associated GI symptoms.

This was confirmed in a randomized, controlled study involving 295 patients with newly diagnosed iron deficiency anemia. Researchers showed that co-administration of Lp299v with high dose iron replacement therapy (IRT; 100 mg daily) resulted in a markedly lower incidence of GI intolerance compared with IRT alone (13.0% vs. 46.5%, *p* < 0.001), as well as substantial reductions in abdominal pain, bloating, nausea, and constipation, and significantly decreased treatment discontinuation rates within the first 30 days (3.6% vs. 15.9%, *p* < 0.001). Importantly, patients receiving Lp299v also exhibited improved iron status markers, including serum iron and transferrin saturation, and greater hemoglobin gains (*p* < 0.001 for all) over the 3-month study period (47).

## 5 Translational applications

The demonstration that Lp299v significantly enhances non-heme iron absorption and improves overall iron status markers in controlled human studies has important translational implications for addressing iron deficiency across diverse populations. Iron deficiency remains the most common micronutrient deficiency globally, disproportionately affecting women of reproductive age, pregnant individuals, children, and populations consuming predominantly plant-based diets, where iron bioavailability is limited. Within this context, the approximately 20–50% relative increase in fractional iron absorption observed with Lp299v supplementation in human trials suggests that incorporation of this probiotic strain into dietary strategies could serve as a practical adjunct to conventional iron fortification and supplementation, particularly where gastrointestinal side effects limit adherence. For example, in women of reproductive age, co-consumption of Lp299v with meals or iron-containing beverages consistently increased absorption by several percentage points, which, when extrapolated over time, may meaningfully improve iron status without increasing iron dose. This has direct relevance for pregnancy and athletic populations, as well, where iron requirements are elevated, and tolerability of iron supplements is often problematic.

From a public health perspective, Lp299v offers flexibility in delivery formats, including suitability for incorporation into beverages, fermented foods, or freeze-dried capsules. This versatility supports implementation across settings spanning low-resource environments to highly industrialized contexts. For instance, integration into fortified staple foods or low-cost functional beverages could enhance iron bioavailability in regions with limited access to animal-source foods, while capsule or functional product formats may be more suitable for individualized supplementation strategies in higher-income settings. Importantly, the ability of Lp299v to enhance iron absorption even in meals already optimized with enhancers such as ascorbic acid indicates additive benefits beyond traditional dietary modifications, making it particularly attractive for vegetarian and vegan populations where dietary inhibitors (such as phytates) limit iron uptake.

Emerging concepts in personalized nutrition further expand the translational potential of Lp299v. Observed inter-individual variability in absorption response suggests that host factors such as baseline microbiota composition may modulate efficacy. This heterogeneity positions Lp299v as a candidate intervention within precision nutrition frameworks, where microbiome-informed analysis could identify individuals most likely to benefit. Such an approach aligns with the broader shift toward tailoring nutritional interventions to individual biological characteristics, representing a potential evolution in the management of micronutrient deficiencies.

Finally, the safety profile and tolerability of Lp299v supports its widespread application, including in vulnerable populations. The strain has been shown to survive gastrointestinal transit and colonize the intestinal mucosa, and it has an established history of safe use in humans in a variety of formats, from capsules to functional foods. Unlike conventional iron supplementation, which can cause GI discomfort and deleteriously alter gut microbiota, Lp299v demonstrates physiologically compatible means of improving iron uptake, potentially mitigating adverse effects associated with unabsorbed luminal iron. These properties—efficacy across dietary contexts, adaptability to diverse delivery platforms, compatibility with global health needs, and potential for integration into personalized nutrition strategies—underscore the translational relevance of strain-specific probiotics, and more specifically Lp299v, as a scalable, adjunctive tool in the global effort to reduce iron deficiency.

## 6 Discussion

With over 2 billion individuals affected, iron deficiency poses a substantial global public health concern. Moreover, women of reproductive age and pregnant women are at particular risk. Current evidence suggests that probiotic strains such as Lp299v could play a role in addressing this global need by serving as a modulator of non-heme iron bioavailability and thus an adjunct strategy for tackling iron deficiency.

Iron absorption is a tightly regulated, multi-step process influenced by luminal chemistry, epithelial transport capacity, and systemic regulatory mechanisms such as hepcidin. Microbiota-mediated modulation of these pathways—through luminal acidification, organic acid–mediated chelation, mitigation of dietary inhibitors, upregulation of key reductive enzymes such as DCYTB, and improvements in epithelial integrity and inflammatory response—provides a mechanistic framework explaining how Lp299v enhances iron status. These mechanisms are supported by *in vitro* studies demonstrating altered iron speciation and increased epithelial reductive capacity, as well as by human isotope studies showing reproducible, clinically meaningful increases in fractional iron absorption across diverse dietary contexts.

Importantly, these mechanistic and acute absorption findings are complemented by randomized clinical data indicating that improved bioavailability can translate into stabilization or improvement of iron status, particularly in populations with elevated requirements or marginal stores. The consistency of enhanced absorption underscores the role of Lp299v as a bioavailability enhancer rather than a direct replacement for iron intake. This distinction is critical, as it positions Lp299v as a complementary strategy that may increase the efficiency of existing dietary and supplementation approaches, potentially enabling lower iron doses and improving tolerability.

Despite numerous preclinical and clinical studies involving Lp299v demonstrating iron absorption effects, they are not without limitations. The (48, 49), and (51), studies showed consistently increased absorption of iron with Lp299v intake; however, the (50), study showed no significant benefit, which may point to complexities in dietary matrices as well as in which state the bacteria is consumed (46, 47, 52, 54), and (56), showed longitudinal iron status effects with Lp299v intake; however, the (57), study showed no significant benefit, which may be related to intervention dose and duration considerations in the pediatric population. Additionally, while some of the clinical trials involve a large number of subjects (e.g. (46), *n* = 326, and (47), *n* = 295), others are smaller in size (e.g. (54), *n* = 39 (56); *n* = 20; and (52), *n* = 13). Some of the smaller studies showed statistically significant endpoints, but in other cases the differences did not reach significance, possibly due to the studies being underpowered. The overall heterogeneity of Lp299v studies complicates inter-study comparison and warrants further investigation into the vagaries of microbiome-driven iron absorption. However, this heterogeneity reflects real-world settings where different dietary matrices and populations have different outcomes.

## 7 Conclusion

In summary, the present paper describes the major health challenge of iron deficiency, the need for additional treatment strategies, and how probiotics, in this case the strain Lp299v, presents one potential approach to addressing this issue. Mechanistic explanations of how this specific strain may affect iron absorption are discussed and merged with the pre-clinical and clinical findings. The overall conclusion based on available evidence suggests that Lp299v offers a scientifically grounded, mechanistically supported, and clinically relevant approach to improving iron bioavailability and overall iron status. By integrating microbiome-based strategies with clinically evidenced probiotic strains, and established nutritional interventions, the current paper offers a novel avenue to address the persistent global burden of iron deficiency.
